# Celsr1 and Celsr2 exhibit distinct adhesive interactions and contributions to planar cell polarity

**DOI:** 10.3389/fcell.2022.1064907

**Published:** 2023-01-12

**Authors:** Lena P. Basta, Parijat Sil, Rebecca A. Jones, Katherine A. Little, Gabriela Hayward-Lara, Danelle Devenport

**Affiliations:** ^1^ Department of Molecular Biology, Princeton University, Princeton, NJ, United States; ^2^ Current Affiliation. University of Pennsylvania, Philadelphia, PA, United States

**Keywords:** planar cell polarity, PCP, epidermis, CELSR1, CELSR2, cadherin, adhesion GPCR, hair follicle

## Abstract

Cadherin EGF LAG seven-pass G-type receptor (Celsr) proteins 1-3 comprise a subgroup of adhesion GPCRs whose functions range from planar cell polarity (PCP) signaling to axon pathfinding and ciliogenesis. Like its *Drosophila* ortholog, Flamingo, mammalian Celsr1 is a core component of the PCP pathway, which, among other roles, is responsible for the coordinated alignment of hair follicles across the skin surface. Although the role of Celsr1 in epidermal planar polarity is well established, the contribution of the other major epidermally expressed Celsr protein, Celsr2, has not been investigated. Here, using two new CRISPR/Cas9-targeted Celsr1 and Celsr2 knockout mouse lines, we define the relative contributions of Celsr1 and Celsr2 to PCP establishment in the skin. We find that Celsr1 is the major Celsr family member involved in epidermal PCP. Removal of Celsr1 function alone abolishes PCP protein asymmetry and hair follicle polarization, whereas epidermal PCP is unaffected by loss of Celsr2. Further, elimination of both Celsr proteins only minimally enhances the *Celsr1* ^*−/−*^ phenotype. Using FRAP and junctional enrichment assays to measure differences in Celsr1 and Celsr2 adhesive interactions, we find that compared to Celsr1, which stably enriches at junctional interfaces, Celsr2 is much less efficiently recruited to and immobilized at junctions. As the two proteins seem equivalent in their ability to interact with core PCP proteins Vangl2 and Fz6, we suggest that perhaps differences in homophilic adhesion contribute to the differential involvement of Celsr1 and Celsr2 in epidermal PCP.

## Introduction

Cadherin EGF LAG seven-pass G-type receptors (Celsr) are atypical cadherins that comprise a subgroup of the adhesion G-protein coupled receptors (GPCRs) (Langenhan et al., 2013; Krishnan et al., 2016). They are distinguished by their large ectodomains consisting of N-terminal cadherin repeats that engage in homophilic adhesion (Wang et al., 2014; Goffinet and Tissir, 2017). Vertebrates have 3 Celsr genes, *Celsr1-3*, that are orthologous to *Drosophila Flamingo* (*Fmi, aka Starry night; Stan*), which is best known for its function in planar cell polarity (PCP), a molecular pathway through which cellular polarity coordinately aligns along an epithelial plane (Boutin et al., 2012; Tissir and Goffinet, 2013; Goffinet and Tissir, 2017). *Celsr* genes are crucial for embryonic development in vertebrates and their functions range from establishment of epithelial planar cell polarity to neural pathfinding and ciliogenesis (Feng et al., 2012; Tissir and Goffinet, 2013; Goffinet and Tissir, 2017). Mutations in mouse *Celsr1*, for example, cause severe defects in neural tube closure (Curtin et al., 2003), and *Celsr2* mutations cause defects in motile cilia formation leading to fatal hydrocephalus (Tissir et al., 2010). In humans, several Celsr1 variants associated with neural tube defects have been identified, implicating these proteins in human development and disease (Allache et al., 2012; Robinson et al., 2012; Lei et al., 2014; Qiao et al., 2016). The expansion of the Celsr subfamily in vertebrates has likely allowed each homolog to evolve different functions, but the overlapping and distinct functions of Celsr proteins are only partially known. Moreover, molecular details of Celsr regulation and function are lacking.

**FIGURE 1:**
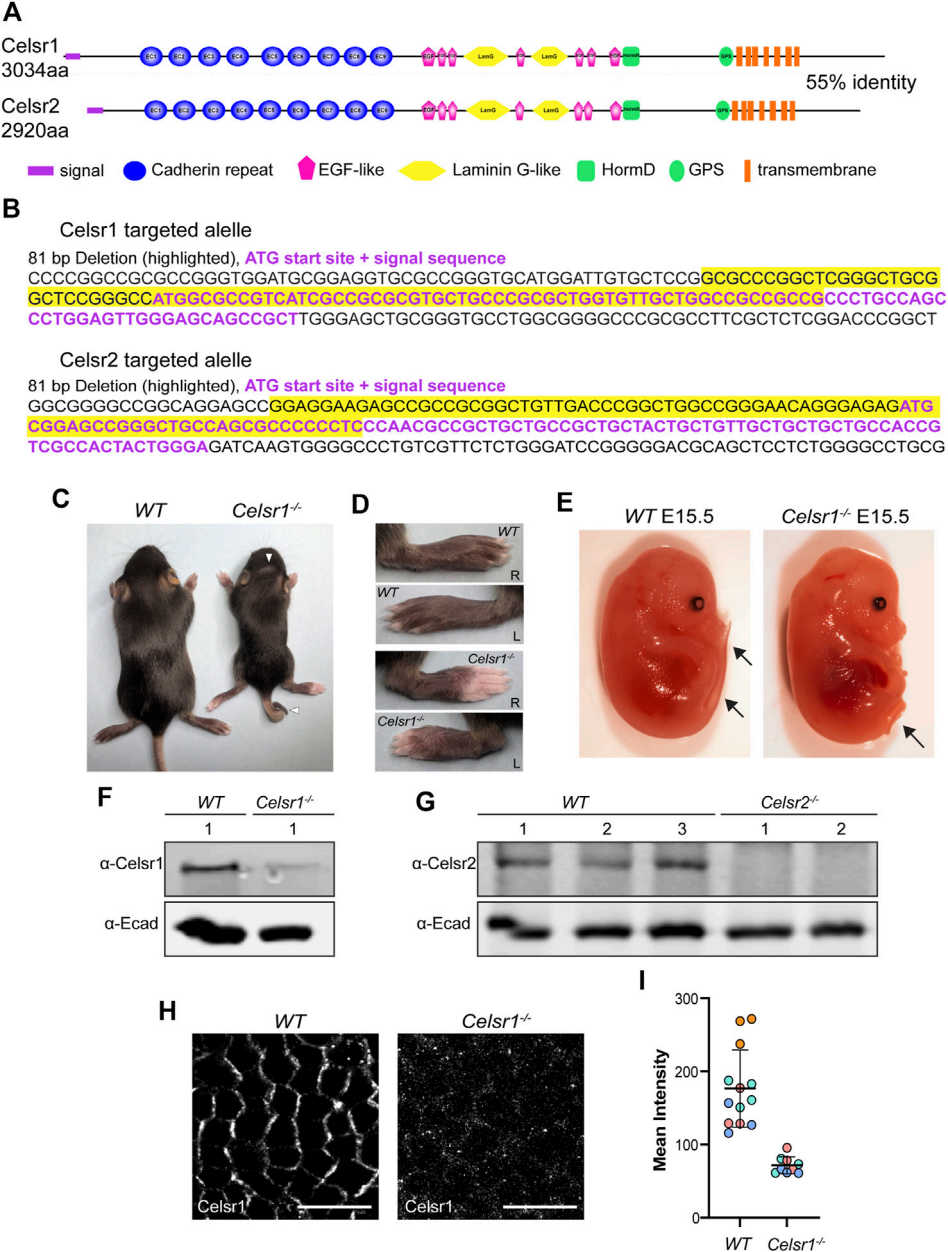
Generation of Celsr1 and Celsr2 loss-of function mutant mice by CRISPR/Cas9. **(A)** Schematic representation of Celsr1 and Celsr2 protein domains. The two proteins are 55% identical in amino acid sequence and have the same overall domain organization. **(B)** CRISPR-Cas9 targeting of *Celsr1* and *Celsr2* genomic loci. Guide RNAs were targeted to the sequence encoding the signal peptide for each of *Celsr1* and *Celsr2*. The resulting targeted alleles are shown with the ATG and signal sequence in purple font and deleted sequences highlighted in yellow. **(C)** *Celsr1* ^*−/−*^ and wild type (*WT*) littermate at P12. Note curly tail and whorled hair pattern on the head of *Celsr1* ^*−/−*^ homozygote. **(D)** Left and right paws of *Celsr1* ^*−/−*^ and *WT* littermate at P12. *Celsr1* ^*−/−*^ homozygotes exhibit prominent hair whorl on each paw. **(E)** *Celsr1* ^*−/−*^ and *WT* littermate embryos at E15.5. *Celsr1* ^*−/−*^ homozygotes display curly tail. **(F)** Western blot of epidermal lysates from WT and *Celsr1* ^*−/−*^ P0-P3 backskins with anti-Celsr1 antibody. **(G)** Western blot of epidermal lysates from three individual *WT* and two individual *Celsr2* ^*−/−*^ P0-P3 pups with anti-Celsr2 antibody. **(H)** Confocal immunofluorescence image of whole mount epidermis from E15.5 *WT* and *Celsr1* ^*−/−*^ mutant embryos labeled with Celsr1 antibodies. Scale bars: 10 µm. **(I)** Quantification of Celsr1 mean fluorescence intensity in *WT* and *Celsr1* ^*−/−*^ mutant epidermis (n = 3 skin regions from 4 different WT embryos and n = 3 skin regions from 3 different *Celsr1* ^*−/−*^ embryos).

Celsr1-3 are very large (>300KD) proteins composed of nine extracellular cadherin repeats, a series of EGF and LamG repeats, a hormone receptor domain (HormD), a GPCR autoproteolysis-inducing (GAIN) domain followed by seven transmembrane helices and a relatively long (∼300–600aa) cytoplasmic tail (Figure 1A)(Wang et al., 2014; Goffinet and Tissir, 2017). Despite their similar domain organization, mouse Celsr1-3 share only ∼35% amino acid identity. *Celsr1-3* transcripts are widely expressed in the nervous system and epithelial organs and are found in both overlapping and tissue-specific expression patterns (Formstone and Little, 2001; Shima et al., 2002; Tissir et al., 2002). Celsr1 and Celsr2 expression overlaps in many embryonic tissues including the brain, kidneys, lung, and olfactory epithelium, whereas Celsr3 is predominantly found in the nervous system (Shima et al., 2002; Tissir et al., 2002). Functionally, Celsr1 is essential for PCP establishment in several mouse epithelial tissues and is considered one of the “core” PCP components (Curtin et al., 2003; Devenport and Fuchs, 2008; Ravni et al., 2009; Boutin et al., 2014; Shi et al., 2014; Stahley et al., 2021). By contrast, Celsr2 and Celsr3 functions have been studied mainly in the nervous system where they have multiple roles in axon pathfinding and brain wiring (Shima et al., 2004; Tissir et al., 2005; Shima et al., 2007; Zhou et al., 2008; Qu et al., 2010; Boutin et al., 2012; Chai et al., 2014; Qu et al., 2014). Celsr2 and Celsr3 are also involved in biogenesis and planar polarization of motile cilia in ependymal cells (Tissir et al., 2010; Boutin et al., 2014), but it is unclear whether Celsr2 and Celsr3 function more broadly in the core PCP pathway outside the cerebral ventricles. In some contexts, such as in cilia biogenesis and axon extension, Celsr2 and Celsr3 are partially redundant (Qu et al., 2010; Tissir et al., 2010; Qu et al., 2014), whereas in other cases their functions are opposed (Shima et al., 2007). It is not known, however, to what extent Celsr2 or 3 act redundantly with Celsr1 in PCP.

Despite their critical roles in embryo morphogenesis and formation of the nervous system, the molecular details of Celsr function remain poorly understood. Aggregation experiments in non-adherent, cadherin-free cell lines (*Drosophila* S2, A431D, and/or K562 cells) have demonstrated that all three Celsr proteins mediate homophilic adhesion *via* their C-terminal cadherin repeats (Shima et al., 2004; Shima et al., 2007; Stahley et al., 2021). Celsr2 and Celsr3 cadherin repeats activate Celsr-mediated responses in neurons, and homophilic Celsr1 adhesion supports stable junctional recruitment and PCP complex organization (Shima et al., 2007; Stahley et al., 2021). Studies on Celsr1 suggest that, like the classical cadherins, Celsr cadherin repeat domains contain binding sites for both trans-adhesive and cis-clustering interactions (Stahley et al., 2021). Beyond their ability to mediate adhesion, which appears to be key for their function, much about the molecular interactions of Celsr proteins remains unknown. Furthermore, differences in adhesive interactions between the different Celsr proteins have not been rigorously tested.

The mouse epidermis is an ideal model for deciphering the relative contributions of Celsr proteins to PCP establishment. In the skin, the PCP pathway governs the polarization and alignment of body hairs across the skin surface (Guo et al., 2004; Devenport and Fuchs, 2008; Ravni et al., 2009). Core PCP proteins, including Celsr1, are expressed in epidermal basal cells, which are the progenitors that give rise to outer skin layers and hair follicles (Devenport and Fuchs, 2008; Basta et al., 2021). Celsr1 localizes asymmetrically at the junctions of basal cells, where it forms homotypic adhesive interactions between anterior and posterior neighbors (Devenport and Fuchs, 2008; Stahley et al., 2021). Celsr1 physically interacts with the other transmembrane PCP components, Fz6 and Vangl2, and promotes their assembly into heterotypic, intercellular complexes (Devenport and Fuchs, 2008; Stahley et al., 2021). Much of what is currently understood about Celsr1 comes from studies of the *Crash* mutant (*Celsr1* ^*Crsh*^), which displays severe PCP-related defects including neural tube closure failure, misoriented stereocilia in the ear and misaligned hair follicles across the surface of the skin (Curtin et al., 2003; Devenport and Fuchs, 2008). The *Crash* mutation maps to a single amino acid substitution (D1040G) in the ectodomain that disrupts the ability of Celsr1 to form stable, clustered assemblies *via* lateral *cis*-interactions (Curtin et al., 2003; Stahley et al., 2021). As a result, Celsr1 asymmetric localization and hair follicle polarity are disturbed (Devenport and Fuchs, 2008; Stahley et al., 2021). The D1040G mutation does not, however, reduce overall Celsr1 protein levels or its membrane enrichment, nor does it interfere with Fz6 or Vangl2 association (Stahley et al., 2021). Thus, despite its semidominant effects, *Crash* is a hypomorphic allele that impairs some but not all Celsr1 functions. How complete loss of Celsr1 function affects epidermal PCP establishment has not been explored in detail.

Compared with Celsr1, the roles of Celsr2 and Celsr3 in epidermal PCP and other skin functions remain largely unknown. Whereas Celsr2 is expressed in the skin epithelium both at embryonic and postnatal stages, Celsr3 transcripts are not detected (Shima et al., 2002; Sennett et al., 2015). For this reason, we set out to determine the consequences of removing all Celsr function in the skin by generating new CRISPR/Cas9-induced *Celsr1*, *Celsr2,* and *Celsr1,2* double knockout mice. Focusing specifically on the establishment of epidermal PCP in single and double *Celsr* loss-of-function mutants, we find that Celsr1 is the major Celsr family member involved in epidermal PCP. Celsr1 removal alone abolishes PCP protein asymmetry and hair follicle polarization, a dramatic phenotype that is among the most severe epidermal PCP defects that have been previously reported (Cetera et al., 2017). By contrast, asymmetric localization of PCP proteins and hair follicle alignment are mostly unaffected by the loss of Celsr2 alone, and removal of both Celsr proteins only minimally enhances the Celsr1 phenotype. To gain insights into the differences between Celsr1 and Celsr2 adhesive interactions, we performed a series of junctional recruitment and FRAP assays in cultured keratinocytes and found that whereas Celsr1 strongly and stably enriches at the junctional interface *via* homophilic adhesive interactions, Celsr2 is much less efficiently recruited to the junction where it is more mobile and diffusive. The two Celsr proteins are capable of interacting heterotypically in trans and are similar in their ability to recruit Fz6 and Vangl2 to junctions. Together, these data show that Celsr1 and Celsr2 display key differences in their ability to form stable, adhesive assemblies, which may underlie, in part, their divergent functions in mouse embryonic development.

## Results

### Generation of Celsr1 and Celsr2 knockout mice using CRISPR/Cas9 gene targeting

To generate deletion mutations in the *Celsr1* and *Celsr2* genes, we used a conventional CRISPR/Cas9 gene targeting approach to induce double stranded breaks and indels in the *Celsr1* and *Celsr2* genomic loci. Guide RNAs were designed to target Cas9 to the genomic regions encoding the translational start sites and signal sequences of each Celsr gene (Figures 1A,B; Supplementary Figure S1). We reasoned that with this strategy, even if an alternative start codon were present, deletion of the signal sequence should prevent co-translational insertion of the protein into the endoplasmic reticulum and result in a non-functional protein product. After screening and sequencing several different mutations that had undergone germ line transmission, two alleles were selected for propagation and backcrossed to establish heterozygous mouse lines. *Celsr1* ^*<em1Ddev>*^ harbors an 81 base pair deletion that includes the translation start site and the first 17 codons of the 29 amino acid signal sequence (Figure 1B; Supplementary Figure S1). *Celsr2* ^*<em1Ddev>*^ also harbors an 81 base pair deletion that includes the start codon and the first 9 codons of the signal sequence (Figure 1B; Supplementary Figure S1).

*Celsr1* ^*<em1Ddev>/<em1Ddev>*^ homozygotes (referred to as *Celsr1* ^*−/−*^ hereafter) were recovered at Mendelian ratios but were smaller and weaker than their heterozygous and wild-type littermates. These animals also displayed curly tails, head shaking behaviors and whorled hair patterns with variable penetrance (Figures 1C,D). Homozygous *Celsr1* ^*−/−*^ embryos displayed curly tails (Figure 1E) and on occasion, neural tube defects. These phenotypes are similar to those reported for a different Celsr1 null mutant and are consistent with defects in the PCP pathway in which Celsr1 is known to function (Ravni et al., 2009). Homozygous *Celsr2* ^*<em1Ddev>*^ mutant animals (referred to as *Celsr2* ^*−/−*^ hereafter) were both viable and fertile did not display any overt morphological defects at birth. However, many developed hydrocephalus postnatally (not shown), also in line with prior reports of a different Celsr2 allele (Tissir et al., 2010).

Using western blots with antibodies against Celsr1 we detected a ∼300KD protein band in epidermal lysates prepared from wild-type embryos (Figure 1F). This band was strongly diminished in lysates from *Celsr1* ^*−/−*^ epidermis suggesting the Celsr1 protein either fails to be translated or is degraded. However, our ability to detect even wild-type Celsr1 by western blot was variable, and a faint band of similar size was still detectable in *Celsr1* ^*−/−*^ lysates, so we turned to immunofluorescence to confirm the protein reduction in *Celsr1* ^*−/−*^ mutants. In wild-type embryonic epidermis at E15.5, Celsr1 is expressed in the basal layer of the skin epithelium where it localizes asymmetrically to anterior-posterior junctions (Figure 1H). By contrast, Celsr1 immunofluorescence was strongly reduced in *Celsr1* ^*−/−*^ embryos (Figure 1I) and what fluorescent signal remained was diffuse and unlocalized (Figure 1H), further suggesting *Celsr1* ^*−/−*^ mutants do not make functional protein product.

Western blots with a Celsr2 antibody also detected a ∼300KD band in lysates from control epidermis, which was not present in lysates from homozygous *Celsr2* ^*−/−*^ mice (Figure 1G). Given the genomic locations of the mutations together with phenotypic, western blotting and immunofluorescence data, we conclude that both *Celsr1* ^*<em1Ddev>*^ and *Celsr2* ^*<em1Ddev>*^ mutant alleles are likely to be protein null. Though we cannot rule out the possibility that cryptic start sites downstream of the *Celsr1* and *Celsr2* deletions may generate partial protein products, we predict these peptides would lack an N-terminal signal sequence and be targeted for degradation.

### Celsr1, but not Celsr2, is required for hair follicle polarization

**FIGURE 2:**
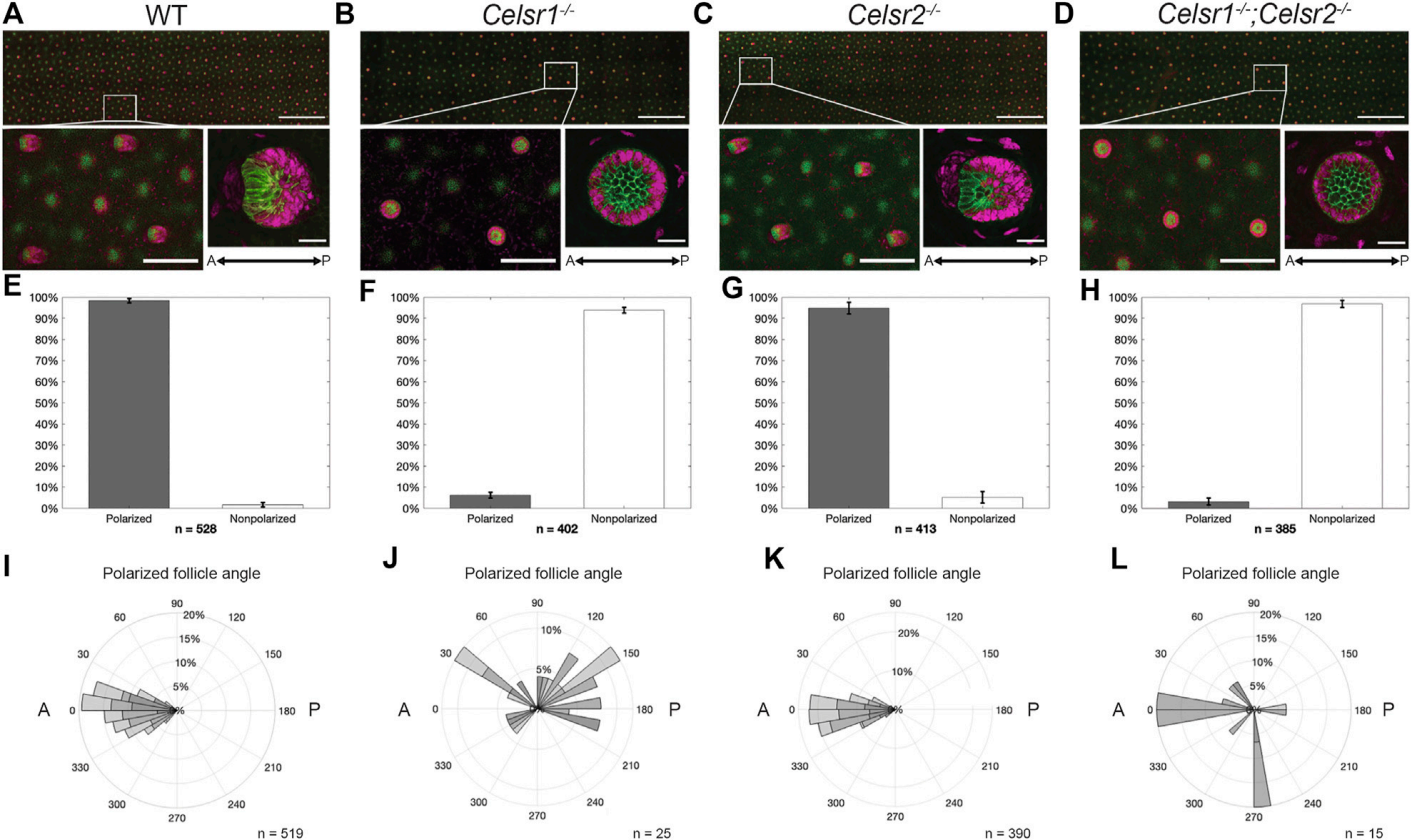
Celsr1, but not Celsr2, is necessary for correct asymmetric orientation of developing hair follicles. **(A)** Average intensity projection of *WT* embryonic back skin at E15.5, labelled for P-cadherin (green) and Sox9 (magenta). White box denotes zoomed in region shown below, left. Average intensity projection of a typical *WT* hair follicle imaged at higher mag (below, right). Scale bars: 1000, 200, and 25 µm, respectively. Anterior is to the left. **(B–D)** As for **(A)**, except *Celsr1* ^*−/−*^, *Celsr2* ^*−/−*^ and *Celsr1* ^*−/−*^ *;Celsr2* ^*−/−*^ respectively. **(E)** Bar chart showing cumulative percentage of polarized (grey bar) vs. non-polarized (white bar) hair follicles in n = 3 E15.5 back skins from 3 different embryos. n in figure represents total number of follicles analyzed. Error bars = SEM. **(F–H)** As for **(E)**, except *Celsr1* ^*−/−*^, *Celsr2* ^*−/−*^ and *Celsr1* ^*−/−*^ *;Celsr2* ^*−/−*^ respectively. **(I)** Rose plot of polarized follicles in **(E)** showing the angle of orientation, with anterior = 0° and posterior = 180°. Shaded areas in bars represent relative contribution of each replicate (n = 3 backskins from 3 different embryos), with n in figure representing total number of polarized hair follicles analyzed. **(J–L)** As for **(I)**, except *Celsr1* ^*−/−*^, *Celsr2* ^*−/−*^ and *Celsr1* ^*−/−*^ *;Celsr2* ^*−/−*^ respectively.

Correct anterior-posterior (A-P) orientation of mammalian hair follicles relies upon core PCP pathway function. Mutations in Fz6, Vangl2, and Celsr1 have all been previously shown to disrupt the asymmetric morphogenesis and coordinated alignment of hair follicles (Guo et al., 2004; Devenport and Fuchs, 2008; Ravni et al., 2009; Chang et al., 2016; Cetera et al., 2017). However, much of what we know about Celsr1 function in the skin comes from examination of the Celsr1 *Crash* mutant, a point mutation that disrupts Celsr1 asymmetry, but does not reduce overall protein levels at epidermal cell junctions (Stahley et al., 2021). The *Celsr1* ^*−/−*^ mouse model we have generated differs from the *Crash* mutant in that no Celsr1 protein is detectable at epidermal cell junctions (Figure 1H) allowing us to determine the phenotypic consequences of a Celsr1 loss-of-function mutant. To investigate this, we labeled E15.5 backskins with P-cadherin and Sox9 antibodies, which mark distinct populations of progenitor cells positioned on the anterior or the posterior of polarized hair follicles, respectively (Figure 2A)(Cetera et al., 2018). In agreement with previously reported follicle polarity defects observed with other Celsr1 alleles, hair follicle orientation in the *Celsr1* ^*−/−*^ embryonic backskins was severely disrupted. Instead of polarizing along the A-P axis and growing toward the anterior, most hair follicles grew straight down, vertically into the dermis, clearly identifiable as a ‘bicycle wheel’ like ring of Sox9 expression surrounding a central cluster of P-cadherin expressing cells (Figure 2B). To quantify both the number and orientation of polarized hair follicles across entire backskins, we used an automated segmentation and follicle angle calculation algorithm, followed by *ad hoc* hand correction (see Methods). Whereas wild-type hair follicles were robustly polarized and grew in an anterior direction (Figures 2E,H), over 90% of hair follicles in the *Celsr1* ^*−/−*^ embryonic backskins were unpolarized and displayed vertically-oriented growth. The few *Celsr1* ^*−/−*^ follicles that did display PCad-Sox9 asymmetry were oriented randomly relative to the A-P axis (Figures 2F,I).

In contrast to Celsr1, little is known about the role of Celsr2 in the mammalian epidermis. *Celsr2* mRNA is expressed in both the epidermis and the placode (Sennett et al., 2015). As such, we next asked whether loss of Celsr2 affects hair follicle orientation in a similar way to Celsr1. Unlike in *Celsr1* ^*−/−*^ embryos, hair follicles in *Celsr2* ^*−/−*^ embryos displayed proper A-P orientation and were indistinguishable from wild type, suggesting that Celsr2 is dispensable for proper hair follicle orientation in mouse embryonic backskin (Figures 2C,G,J).

We next asked whether removal of Celsr2 would enhance the hair follicle phenotype observed in *Celsr1* ^*−/−*^ embryos. To do so, we crossed the *Celsr1* ^*−/+*^ and *Celsr2* ^*−/−*^ mice to generate homozygous, double mutant embryos (*Celsr1* ^*−/−*^;*Celsr2* ^*−/−*^). Backskins from E15.5 *Celsr1* ^*−/−*^;*Celsr2* ^*−/−*^ embryos were then labeled with Sox9 and P-cadherin antibodies and hair follicle polarity was analyzed as above. The hair follicle phenotype of *Celsr1* ^*−/−*^;*Celsr2* ^*−/−*^ embryos was indistinguishable from *Celsr1* ^*−/−*^ embryos, further signifying that Celsr1, but not Celsr2 is required for proper hair follicle orientation (Figures 2D,H,K). We conclude from these data that Celsr1 is the major core PCP cadherin functioning in the epidermis.

### Celsr1 and, to a lesser extent, Celsr2 are required for asymmetric localization of core PCP components

Hair follicle polarization relies upon the asymmetric distribution of core PCP proteins at the intercellular junctions of epidermal basal cells (Devenport and Fuchs, 2008; Cetera et al., 2017; Cetera et al., 2018). Celsr1 localizes to the anterior and posterior junctions of each cell, where it colocalizes with Vangl2 and Fz6, respectively (Devenport and Fuchs, 2008; Basta et al., 2021; Stahley et al., 2021). Based on polarity analysis in the skin of Celsr1 *Crash* mutant embryos, we know that PCP asymmetry relies on proper Celsr1 function. The Celsr1 *Crash* mutant mouse (*Celsr1* ^*Crsh*^), harbors a single amino acid substitution (D1040G) that disrupts the ability of Celsr1 to form lateral *cis*-interactions and as a result, Celsr1 clustering, junctional stability and asymmetry are all impaired (Stahley et al., 2021). In *Drosophila*, Fz and Vang are lost from apical junctions in Fmi mutants suggesting that, in addition to promoting their asymmetric localization, Fmi recruits and/or stabilizes Fz and Vang at cell junctions (Strutt, 2001; Bastock et al., 2003; Chen et al., 2008). Whether Celsr proteins perform a similar function in mammals is unknown as this has not been tested in loss-of-function mutants.

**FIGURE 3:**
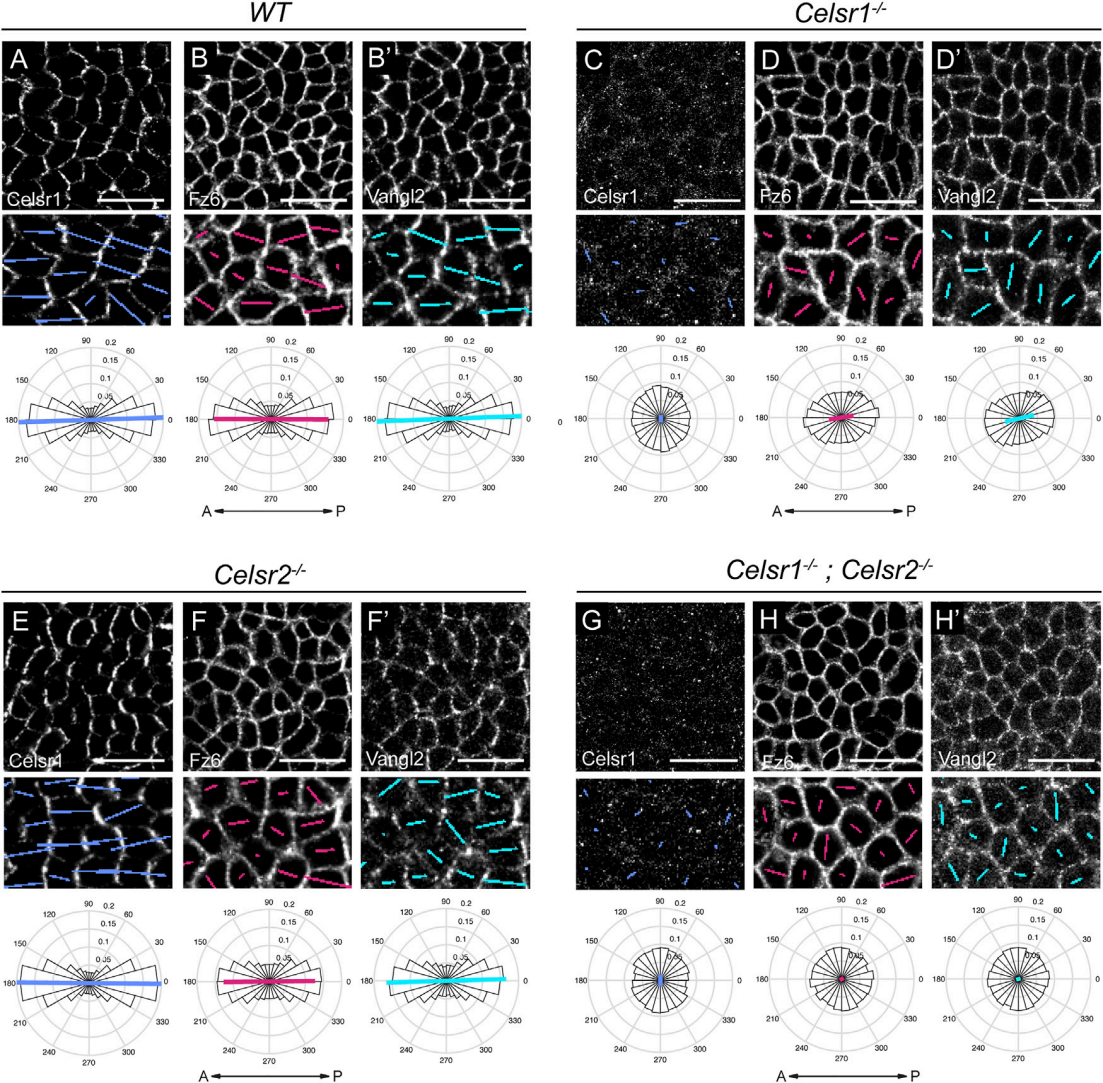
Loss of core PCP protein asymmetry in the epidermis of *Celsr1* ^*−/−*^ and *Celsr1* ^*−/−*^;*Celsr2* ^*−/−*^ double mutants. **(A–H)** Representative planar views of the basal layer of the interfollicular epidermis at E15.5 showing Celsr1, Fz6 and Vangl2 distribution as detected by immunofluorescence. Anterior is to the left. Scale bar: 20 µm. Magnified areas below are overlaid with colored lines representing the axis (line angle) and magnitude (line length) of polarity. Quantification of polarity distributions are displayed below on circular histograms. **(A-B′)** Celsr1 **(A)**, Fz6 **(B)** and Vangl2 (B′) in *WT* embryos, n = 11,951 basal cells, 3 embryos. **(C-D′)** Celsr1 **(C)**, Fz6 **(D)**, and Vangl2 (D′) in *Celsr1* ^*−/−*^ embryos, n = 11,629 basal cells, 3 embryos. **(E-F′)** Celsr1 **(E)**, Fz6 **(F)** and Vangl2 (F′) in *Celsr2* ^*−/−*^ embryos, n = 12,099 basal cells, 3 embryos. **(G-H′)** Celsr1 **(G)**, Fz6 **(H)** and Vangl2 (H′) in *Celsr1* ^*−/−*^ *; Celsr2* ^*−/−*^ embryos, n = 9,064 basal cells, 3 embryos.

To test whether Celsr1 and Celsr2 are required for the recruitment and/or polarization of core PCP components, we measured the orientation and magnitude (nematic order) of Celsr1, Fz6 and Vangl2 asymmetry along cell junctions. To do this, we imaged whole mount E15.5 backskins labeled with antibodies against Celsr1, Vangl2 and Fz6. Automated segmentation of epithelial edges was performed using E-Cadherin or P-Cadherin as a junctional marker (Aigouy et al., 2016). The nematic order of PCP protein fluorescent intensities was measured using QuantPolarity software and displayed on radial histograms (Tan et al., 2021). In wild-type control epidermis, Celsr1, Fz6 and Vangl2 were all enriched along A-P junctions and depleted from M-L junctions, and this asymmetry was highly aligned along the A-P axis (Figures 3A–B’). As expected from previous studies on *Celsr1* ^*Crsh*^ mutant mice (Devenport and Fuchs, 2008; Stahley et al., 2021), we found that PCP protein asymmetries were dramatically reduced in *Celsr1* ^*−/−*^ mutants. Celsr1 immunofluorescence was strongly diminished at cell junctions (Figure 3C), and both Fz6 and Vangl2 were distributed more uniformly around the periphery of basal cells compared to controls (Figures 3D–D’). By contrast, the asymmetry of all three core PCP proteins was mostly unaffected in *Celsr2* ^*−/−*^ mutants. Although Fz6 and Vangl2 localization appeared less sharply concentrated at junctions, quantification of asymmetry showed the magnitude and orientation of their polarity were comparable to wild-type controls (Figures 3E–F’), consistent with the normal alignment of hair follicles observed in *Celsr2* ^*−/−*^ mutants (Figure 2C). Fz6 and Vangl2 asymmetries were more severely reduced in *Celsr1* ^*−/−*^ *; Celsr2* ^*−/−*^ double mutant embryos compared to *Celsr1* ^*−/−*^ single mutants (Figures 3H–H’), suggesting that in the absence of Celsr1, Celsr2 does provide a modest contribution to PCP protein localization. Notably, despite the loss of Fz6 and Vangl2 polarization in *Celsr1* ^*−/−*^ *; Celsr2* ^*−/−*^ double mutants, we did not observe appreciable reduction in membrane recruitment of either Vangl2 or Fz6 (Figures 3H–H’). This suggests that unlike Fmi in *Drosophila*, Celsr proteins are not needed to traffic and/or retain Fz and Vangl to cell junctions, but rather to organize them into polarized junctional assemblies.

### Differential stability of Celsr1 and Celsr2 homotypic adhesions at cell junctions

**FIGURE 4:**
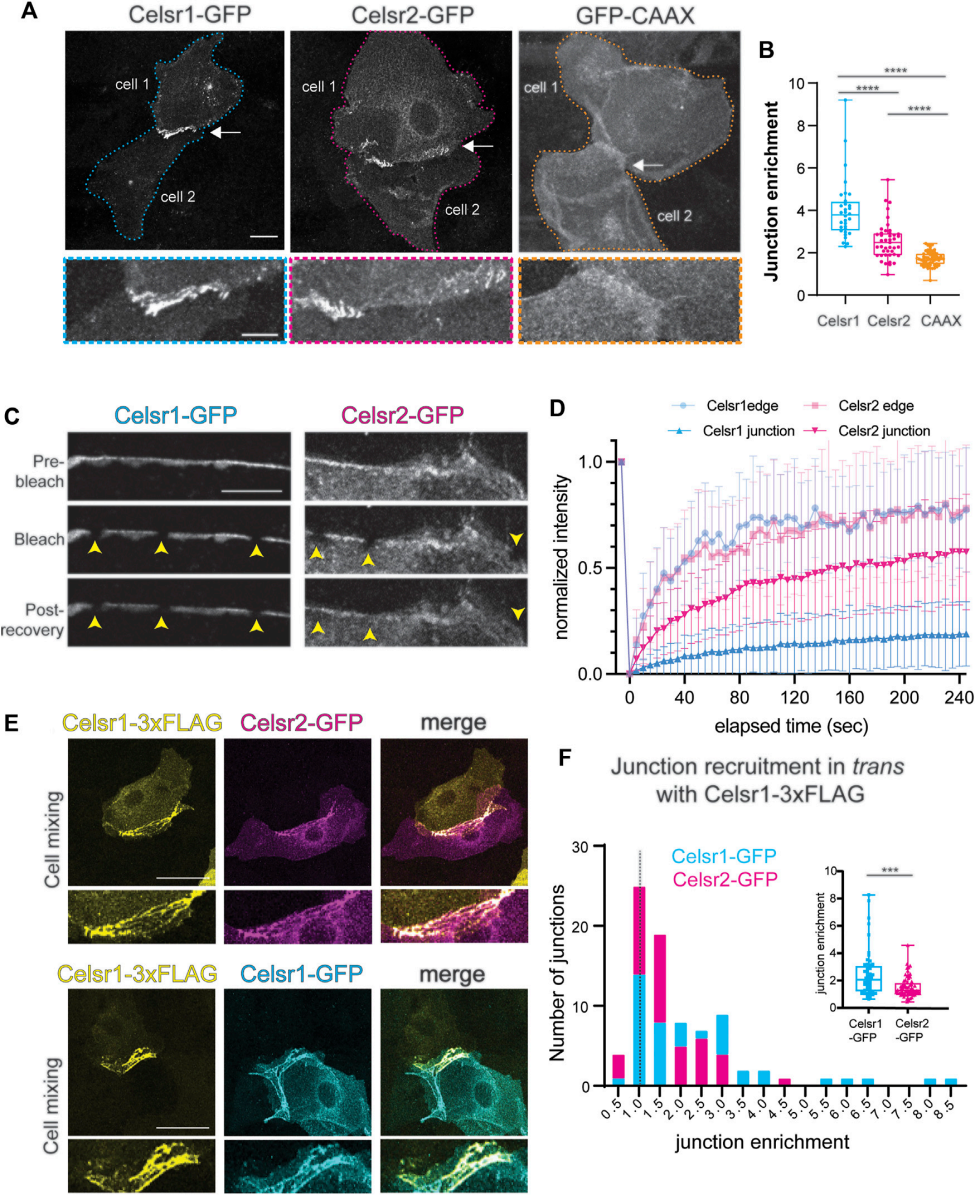
Celsr2 enriches at cell-cell junctions by homotypic interactions less efficiently than Celsr1. **(A)** Representative images of cell pairs expressing Celsr1-GFP, Celsr2-GFP or GFP-CAAX as indicated. Bottom panels show zoomed in junctional regions. Note the stronger enrichment of Celsr1-GFP at junctions compared to Celsr2-GFP and both isoforms are significantly more enriched at junctions compared to a non-junctional plasma membrane marker GFP-CAAX. Scale bars 20 µm (top panel) and 10 µm (bottom panel). **(B)** Plot of the junctional enrichment score (ratio of junctional mean intensity to the mean intensity of the cell pair). n = 32 Celsr1-GFP junctions, n = 43 Celsr2-GFP junctions, n = 60 GFP-CAAX junctions. Kolmogorov-Smirnov test *p* < 0.0001. Data pooled from two independent experiments where each experiment reflects the represented trend. **(C)** Fluorescence Recovery After Photobleaching (FRAP) of junctional Celsr1-GFP and Celsr2-GFP. Shown are representative images of the junctional region between cell pairs expressing Celsr1-GFP or Celsr2-GFP before and after bleaching as indicated. Bleached ROIs are marked by yellow arrowheads. **(D)** FRAP recovery plots. Shown is the normalized mean intensity with standard deviations of the bleach and recovery profiles plotted *versus* time for Celsr1-GFP (blue) and Celsr2-GFP (magenta) at junctions (in bold) and free cell edges that are not juxtaposed to a transfected cell (in lighter shade). (n = 36 ROIs for Celsr1 edge, 38 ROIs for Celsr2 edge, 78 ROIs for Celsr1 junctions and 75 ROIs for Celsr2 junctions). Data pooled from two independent experiments for cell edge measurements and three independent experiments for junction measurements. **(E)** Cell mixing experiment between cells expressing Celsr1-3xFLAG and Celsr2-GFP (top panels) or Celsr1-3xFLAG and Celsr1-GFP (bottom panels). Images show cell pairs forming heterotypic junctions. Celsr1-3xFLAG appears to enrich with both Celsr1-GFP and Celsr2-GFP, *in trans*, across cell-cell junctions. **(F)** Histogram depicting the frequency of Celsr1-3xFLAG: Celsr1-GFP and Celsr1-3xFLAG::Celsr2-GFP junctions across the range of junction enrichment ratios obtained for Celsr1-GFP and Celsr2-GFP, respectively. Inset shows box plot for the junction enrichment values of Celsr1-GFP and Celsr2-GFP. n = 56 Celsr1-GFP junctions and n = 64 Celsr2-GFP junctions. Kolmogorov-Smirnov test, *p* = 0.0004. Data pooled from two independent experiments.

Our data thus far demonstrate that, despite their relatively similar levels and patterns of expression in the skin (Sennett et al., 2015; and Allen Mouse Brain Atlas, developingmouse.brain-map.org/experiment/show/100057665; developingmouse.brain-map.org/experiment/show/100055676) Celsr1 and Celsr2 contribute very differently to PCP function in the epidermis. Celsr1 plays a far more essential role whereas Celsr2 is largely dispensable. We hypothesized that perhaps the two epidermally-expressed Celsr proteins display different adhesive properties and/or abilities to interact with known PCP partners, which might explain their different contributions to PCP function. To explore this, we first compared the homophilic interactions of Celsr1 and Celsr2 in a junctional enrichment assay in cultured keratinocytes. Celsr1-GFP or Celsr2-GFP constructs were transiently transfected into primary mouse keratinocytes that were derived from the backskins of *Celsr1* ^*−/−*^ *; Celsr2* ^*−/−*^ double mutant embryos generated in this study so that the only functional Celsr proteins were the introduced GFP-tagged proteins. Adhesive monolayers were induced by increasing the calcium concentration in the media to allow for cadherin-based cell-cell adhesion. In this assay, Celsr1-GFP becomes selectively enriched at the interface between two Celsr1-GFP expressing cells in a calcium- and ectodomain-dependent manner indicating the enrichment is a result of cadherin-domain mediated homophilic interactions between Celsr1 proteins on adjacent cells (Devenport and Fuchs, 2008) (Figure 4A, Supplementary Figure S4). To measure the degree of enrichment, we calculated a junctional enrichment score (JE) for cell pairs expressing Celsr-GFP (ratio of the mean junctional intensity to the mean intensity of the cell pair) (Figure 4B). Membrane associated GFP-CAAX was used as a negative control for the baseline enrichment observed when membranes of adjacent cells overlap (JE^GFP−CAAX^< 2) (Figures 4A,B). As expected from prior studies, Celsr1 was strongly enriched at the junctional interfaces between expressing cells with an average enrichment score of approximately 4 (Stahley et al., 2021) (Figures 4A,B, Supplementary Figure S4). Celsr2-GFP localization, by contrast, was significantly more diffuse across cell pairs (Figures 4A,B, Supplementary Figure S4; mean JE^Celsr2−GFP^ ∼2.5) but was still enriched to a greater extent than the GFP-CAAX baseline (mean JE < 2) (Figures 4A,B). This indicates that Celsr2 does interact homophilically *in trans* in epithelial cells. This result is consistent with Celsr2’s ability to mediate aggregation in S2 cells (Shima et al., 2004). Interestingly, when cells transfected with Celsr1-3xFLAG were mixed with Celsr2-GFP expressing cells, they formed heterotypic junctions *in trans* between mixed cell pairs (Figure 4E). The enrichment of Celsr2-GFP with Celsr1-3xFLAG at Celsr1/2 heterotypic junctions was, however, lower than the enrichment of Celsr1-GFP with Celsr1-3xFLAG (Figures 4E,F). This observation indicates that despite the differences in Celsr1 and Celsr2 homotypic junctions, their ectodomains are similar enough to interact heterotypically.

The enrichment of cell-cell adhesion proteins to junctions correlates with their relative immobility within the interfacial membrane (Stahley et al., 2021). We have previously shown that Celsr1 is remarkably stable at cell junctions, where it is more immobile than junctional E-Cadherin (Aw et al., 2016; Stahley et al., 2021). To determine if the comparatively low enrichment of Celsr2 at homotypic interfaces is correlated with a greater mobility in the membrane, we performed FRAP assays in cell pairs expressing Celsr1-GFP or Celsr2-GFP. Small regions (1um diameter) along the junctions or free edges of Celsr1-GFP or Celsr2-GFP expressing cell pairs were photobleached and imaged continuously over a 4-min recovery period (Figure 4C, Supplementary Figure S4). Fluorescence recovery curves for Celsr1 and Celsr2 were nearly identical in regions near a free edge where the proteins are most likely unbound intercellularly and free to diffuse within the membrane (Figure 4D). By contrast, at junctions the extent of recovery for Celsr1 and Celsr2 were starkly different. Whereas Celsr1-GFP fluorescence was strongly immobilized (immobile fraction ∼80% at junctions *versus* ∼26% at cell edges, estimated from fitted averaged traces) and recovered only minimally over the entire recovery period, the mobility of Celsr2-GFP was comparatively less attenuated at junctions (immobile fraction ∼43% at junctions *versus* ∼28% at cell edges, estimated from fitted averaged traces) (Figure 4D). These data suggest that the adhesive interactions of Celsr proteins at epithelial junctions are not equivalent and that Celsr1 interactions lead to much greater stability and junctional enrichment.

### Fz6 and Vangl2 are recruited to both Celsr1-and Celsr2-homotypic adhesions

**FIGURE 5:**
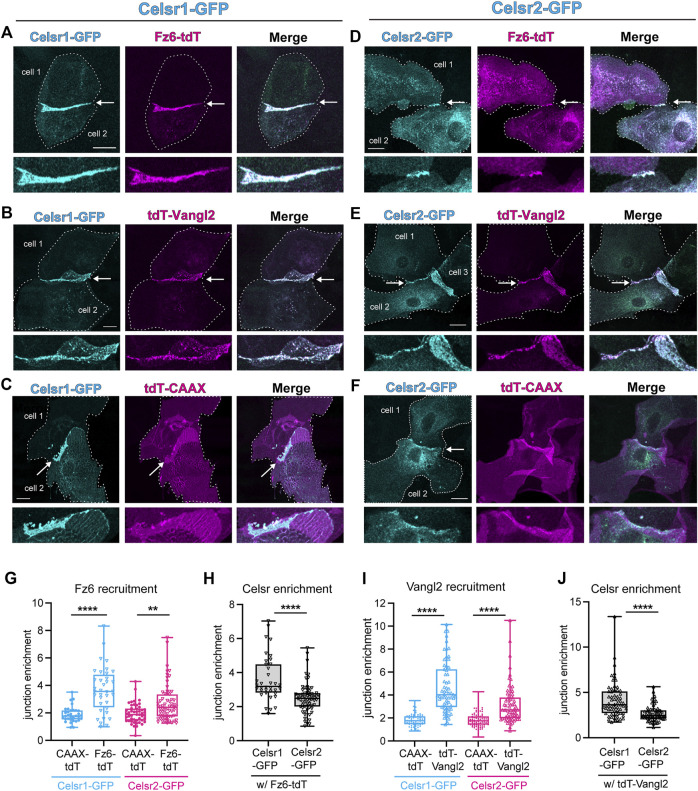
Celsr2 recruits Fz6 and Vangl2 to keratinocyte junctions, similar to Celsr1. **(A–C)** Representative cell pair co-expressing Celsr1-GFP and Fz6-tdTomato **(A)**, tdTomato-Vangl2 **(B)** or a non-junctional membrane marker tdTomato-CAAX **(C)**. Arrowheads mark the junction between 2 cells and a magnified view of the junction is represented below the respective images. Scale bars = 20um. **(D–F)** Representative cell pair co-expressing Celsr2-GFP and Fz6-tdTomato **(D)**, tdTomato-Vangl2 **(E)** and tdTomato-CAAX **(F)**. Arrowheads mark the junction between 2 cells and a magnified view of the junction is represented below the respective images. Scale bars = 20 um. **(G)** Box plots depicting junction enrichment ratios for Fz6-tdTomato compared to tdTomato-CAAX when co-expressed with Celsr1-GFP or Celsr2-GFP (n = 33 for Celsr1-tdTomato CAAX, n = 49 for Celsr2-tdTomato-CAAX, n = 36 for Celsr1-Fz6-tdTomato, n = 66 for Celsr1-Fz6-tdTomato). **(H)** Box plots depicting junction enrichment values of Celsr1-GFP *versus* Celsr2-GFP in cells co-expressing Fz6-tdTomato. **(I)** Box plots depicting junction enrichment ratios for tdTomato-Vangl2 compared to tdTomato-CAAX when co-expressed with Celsr1-GFP or Celsr2-GFP (n = 66- Celsr1-tdTomatoCAAX, n = 87-Celsr2 tdTomatoCAAX, n = 63-Celsr1 tdTomatoCAAX, n = 67-Celsr2-tdTomatoCAAX). **(J)** Box plots depicting junction enrichment of Celsr1-GFP and Celsr2-GFP in cells expressing tdTomato-Vangl2. Data pooled from two independent experiments for Fz6-tdTomato and three independent experiments for tdTomato-Vangl2. Kolmogorov-Smirnov tests, ****p < 0.0001, **p = 0.009.

One important function for Celsr1 and *Drosophila* Fmi in PCP establishment is to physically associate with the other transmembrane core components, Fz and Vang, stabilize them at cell junctions, and promote their assembly into asymmetric, intercellular complexes (Harrison et al., 2020; Stahley et al., 2021). Additionally, in *Drosophila*, Fz and Vang positively feed back onto Fmi stability by preventing its endocytic removal from the membrane (Strutt and Strutt, 2008; Strutt et al., 2011). Thus, a difference in Fz and/or Vang association could explain why Celsr1 and Celsr2 display different junctional dynamics and contributions to PCP. To test this hypothesis, we asked whether Celsr2 can redirect Fz6 and Vangl2 to sites of homotypic adhesion in cultured keratinocytes, as a read-out of their association, as we have previously shown for Celsr1 (Devenport and Fuchs, 2008; Stahley et al., 2021). Primary mouse keratinocytes derived from *Celsr1* ^*−/−*^ *; Celsr2* ^*−/−*^ double mutants were co-transfected with Celsr1-GFP or Celsr2-GFP and either Fz6-tdTomato or tdTomato-Vangl2 and a JE score was calculated for co-expressing cell pairs. tdTomato-CAAX was used as a negative control to establish the baseline JE score for a generic membrane marker. As expected, Fz6-tdTomato and tdTomato-Vangl2 were both recruited to sites of Celsr1-GFP localization and became strongly enriched at the junctional interface between co-expressing cell pairs (Figures 5A,B,G,I) (mean JE^tdT−Fz6^ −.5 and mean JE^tdT−Vangl2^ −4.5). Both proteins were significantly enriched compared to negative control tdTomato-CAAX (Figures 5C,G,I) (mean JE^tdT−CAAX^ −2). Fz6-tdTomato and tdTomato-Vangl2 also localized to Celsr2-GFP enriched junctions (Figures 5D,E,G,I), and their JE scores were significantly greater than that of tdTomato-CAAX (Figures 5F,G,I), indicating that Celsr2 is capable of associating with Fz6 and Vangl2 and directing them to sites of homophilic adhesion. However, Fz6-tdTomato and tdTomato-Vangl2 enrichment was significantly lower than when they were co-expressed with Celsr1-GFP (Figures 5G,I). This difference is most likely due to the lower JE of Celsr2 itself when compared to Celsr1 within these same experiments (Figures 4H–J, Supplementary Figure S5) rather than a major difference in the ability of Celsr2 to associate with Fz6 and Vangl2. We conclude from these data that, like Celsr1, Celsr2 can recruit Fz6 and Vangl2 to sites of homophilic adhesion and, by extension, may possibly physically associate with both proteins.

## Discussion

The Celsr subfamily of adhesion GPCRs are essential for embryonic development and formation of the nervous system, yet we are only just beginning to understand the range of functions that these very large adhesive molecules perform and how adhesion contributes to those functions (Wang et al., 2014; Goffinet and Tissir, 2017). Through the generation of new Celsr1 and Celsr2 knockout mouse strains and examination of both single and double mutant embryos, we have determined the contributions of Celsr proteins to embryonic skin development. Because Celsr3 is not expressed in the skin, our data allow us to confidently conclude that Celsr1 is the major cadherin-based adhesion GPCR functioning in epidermal PCP. Further, we can also conclude that the major function of Celsr proteins in skin development is to establish PCP. Other key developmental processes in the skin such as stratification, hair follicle specification, patterning, and downgrowth are largely unaffected when all Celsr function is removed. On a molecular level, we have identified key similarities and differences in the adhesive interactions and dynamics of Celsr1 and Celsr2 proteins at epithelial junctions. The adhesive interfaces of Celsr1 and Celsr2 are similar enough to engage heterotypically and both proteins can recruit Fz6 and Vangl2. Despite these similarities, their dynamics at homotypic adhesive interfaces are markedly different. Celsr1 is largely immobile at the junction whereas Celsr2 can diffuse much more freely. This difference is reflected in the relative enrichment of the two proteins at junctions.

In our prior work, we showed that Celsr1 is very stable at cell junctions, where it has a lower mobility than junctional E-Cadherin (Aw et al., 2016; Stahley et al., 2021). Celsr1’s immobility is in part due to clustering interactions that organize Celsr1 into large punctate assemblies, a property that is severely diminished by the *Crash* mutation located in the membrane proximal cadherin repeats. Although Celsr2 can mediate cell aggregation when expressed in non-adhesive suspension cells (Shima et al., 2004), it is poorly enriched at junctional interfaces in epithelial cells, and the fraction that is recruited to junctions exhibits greater mobility than Celsr1. Whether these adhesive differences can be attributed to differences in the sequence of their N-terminal cadherin repeats is unclear. Celsr1 and Celsr2 share 55% amino acid identity, but it is their C-terminal cytoplasmic tails, rather than their cadherin repeats, that are the most divergent in sequence. Interestingly the residue mutated in the *Crash* allele of Celsr1, D1040, which maps to the linker region between EC8 and EC9, is conserved in Celsr2, and yet the homophilic adhesive properties of Celsr2 much more closely mimic that of the *Celsr1* ^*Crash*^ mutant than wild type Celsr1, suggesting this residue is necessary but not sufficient for Celsr1 stable association at cell junctions. Interestingly, Celsr1, Celsr2 and the Celsr1^Crash^ mutant proteins are all capable of recruiting core PCP proteins Fz6 and Vangl2 to junctions (Stahley et al., 2021), suggesting it is not the presence of these additional PCP proteins at the junction that contributes to Celsr1 stability. Perhaps differences in the affinity of trans-adhesive interactions, in the avidity of cis-adhesive interactions, or in the identity of cytoplasmic binding partners may account for the different homophilic adhesive properties of the two Celsr proteins. Performing adhesion assays with domain swapped Celsr1-Celsr2 chimeric proteins will distinguish whether adhesive differences are due to extracellular, transmembrane or cytoplasmic regions. Although we do not know the molecular basis for these differences in mobility and enrichment, we propose that they have important functional consequences.

Differences in stability of Celsr1 and Celsr2 adhesions could enable the two proteins to perform distinct functions in dynamic *versus* static cellular contexts. Celsr1 is essential for PCP in the skin, inner ear, oviduct and ependymal cells, all of which are relatively static epithelial tissues that assemble robust, asymmetric PCP complexes at cell junctions (Curtin et al., 2003; Devenport and Fuchs, 2008; Boutin et al., 2014; Shi et al., 2014; Duncan et al., 2017). The immobility of homophilic Celsr1 adhesions at these sites is probably essential for PCP asymmetry. In the *Drosophila* wing, PCP asymmetry is strongly correlated with the assembly of PCP components into stable puncta (Strutt et al., 2011; Cho et al., 2015; Strutt et al., 2016). Moreover, in mouse epidermal cells, disruption of Celsr1 lateral clustering interactions prevents the formation of asymmetric PCP complexes (Stahley et al., 2021). Celsr2, by contrast, is important in neuronal migration and axon pathfinding (Shima et al., 2004; Shima et al., 2007; Qu et al., 2010; Feng et al., 2012; Qu et al., 2014), processes that likely require adhesive interactions that can rapidly turnover, and Celsr2 may be better equipped than Celsr1 for such dynamics. Given that Celsr2 can recruit both Vangl2 and Fz6 to adhesive sites suggests it may form more dynamic PCP-like assemblies in neurons than those containing Celsr1 in epithelia. Interestingly, the single Celsr homolog in *Drosophila*, Fmi, functions in epithelial PCP as well as nervous system development, so it can most likely assemble both stable and dynamic adhesions in a cell type-specific manner (Gao et al., 2000; Berger-Muller and Suzuki, 2011; Shimizu et al., 2011). Perhaps different Fmi splice isoforms are expressed in different cell types, or that cell type specific interacting proteins regulate Fmi’s adhesive state.

Despite the similarity in protein domain organization across the 3 Celsrs, it remains unclear if Celsr2 (or Celsr3) can function as a core PCP component in vertebrates, defined in the strict sense of acting at cell junctions to align cell polarity across a tissue plane (Devenport, 2014). Given that Celsr2 exhibits mobility at the junction comparable to the *Crash* mutant of Celsr1, it is possible Celsr2 cannot asymmetrically localize to junctions in epithelia. Unfortunately, due to lack of antibodies that reliably detect endogenous Celsr2 *in vivo*, we do not presently know the localization of Celsr2 in the mouse epidermis. Further, although endogenous levels of Celsr2 cannot replace Celsr1 in epidermal PCP, we do not know whether overexpression of Celsr2 could compensate. Additionally, given its roles in biogenesis and polarization of motile cilia in the brain (Tissir et al., 2010; Boutin et al., 2014), we were open to the possibility that Celsr2 might have a function related to primary cilia in the skin. However, we did not observe phenotypes associated with cilia disruption in the skin of either *Celsr2* ^*−/−*^ or *Celsr1* ^*−/−*^ *; Celsr2* ^*−/−*^ double mutant embryos, such as defects in epidermal thickness due to impaired Notch signaling or defects in hair follicle morphogenesis related to aberrant Shh signaling (Ezratty et al., 2011; Dai et al., 2013; Chen et al., 2015; Yang et al., 2015). Future studies at postnatal stages will determine whether Celsr proteins function beyond PCP in the skin—for example in hair follicle cycling and regeneration or perhaps in the migration and innervation of sensory neurons.

## Experimental procedures

### Generation of mouse lines and breeding

All mouse work was approved by Princeton University’s Institutional Animal Care and Use Committee (IACUC). Mice were housed in an AALAC- accredited facility. Housing, maintenance, and husbandry of animals followed the Guide for the Care and Use of Laboratory Animals and laboratory Animal Welfare Act.

The mice were generated at the Genome Editing Core Facility at Rutgers-Cancer Institute of New Jersey using CRISPR targeting the start site and signal sequence of the Celsr1 and Celsr2 genes simultaneously. Many knockouts were generated and sequenced. Two mouse lines were selected that contained a deletion of 81 bp spanning the start site and beginning of the signal sequence of Celsr1 and Celsr2, respectively. N1 founder mice were outcrossed to C57Bl/6J five times. The new alleles were named *Celsr1* ^*<em1Ddev>*^ and *Celsr2* ^*<em1Ddev>*^, and were maintained as heterozygotes.

Genotyping PCRs were designed to discriminate between WT and knockout lines. To genotype for the *Celsr1* ^*<em1Ddev>*^ allele, a PCR was designed using primers surrounding the 81 bp deletion, resulting in a smaller fragment in the knockout. Similarly, to genotype the *Celsr2* ^*<em1Ddev>*^ allele, PCR primers were designed on either side of the deletion site (Supplementary Figure S1; see Table 1 for primer sequences and expected PCR product sizes).

**TABLE 1:** Product information of key antibodies and reagents used in this study.

PCR	Name of genotyping primer	Genotyping primer sequences	Product size WT animals	Product size knockout animals
*Celsr1* ^*<em1Ddev>*^	Celsr1.FOR	CAA​CTT​GGC​AAA​CTT​TCG​CAA​AGT​G	396 bp	315 bp
	Celsr1.REV	GCG​CGT​GGT​GTC​CAA​CCT​GTA​G		
*Celsr2* ^*<em1Ddev>*^	Celsr2.FOR	CCA​TCT​GGG​TGC​AGG​CCC​AGT​G	350 bp	269 bp
	Celsr2.REV	GTG​TAG​AGC​CAG​AGG​TTC​GAA​GC		

### Western blot analysis

Epidermal lysates were obtained by dissection of backskin from P0- P2 postnatal pups. To extract proteins, backskins were flash frozen in liquid nitrogen and then ground into a powder in a cryomill machine (Retsch,Newtown, PA) for 30 s while frozen using liquid nitrogen. For each backskin, 700 ul of RIPA buffer (Abcam) was added to the ground powder, the samples were vortexed and then incubated on ice for 15 min. Following incubation on ice, the samples were centrifuged at 17,000g for 15 min at 4°C. Supernatants were removed and processed *via* western blot. Standard protocols were performed for western blot- proteins were resolved on a 7.5% SDS gel, transferred to a nitrocellulose membrane (Bio-Rad), and detected using primary antibodies against Celsr1 (Devenport and Fuchs, 2008), Celsr2 (goat, R&D Systems, 1:200), and E-cadherin (rabbit, Cell Signaling, 1:250 or rat, ThermoFisher, 1:1000). IRDye680 and IRDye800 secondary antibodies (LI-COR, 1:10,000) and the LI-COR Odyssey CLx imaging system were used to detect the bands. See Table 2 for full list of antibodies and reagents.

**TABLE 2:** Genotyping details for Celsr1 and Celsr2 including primer sequences and product sizes for WT and knockout animals.

Reagent type (species) or resource	Designation	Source or reference	Identifiers	Additional information
Antibody	Anti-Celsr1 (Guinea pig polyclonal)	Devenport and Fuchs (2008)		1:1000 (tissue)
				1:2000 (keratinocytes)
Antibody	Anti-Celsr1 (rabbit polyclonal)	Millipore Sigma	ABT119	1:1000 (western blot)
Antibody	Anti-E-cadherin (rabbit monoclonal)	Cell Signaling	Cat #3195	1:250
Antibody	Anti-Celsr2 (goat)	R&D systems	Cat#AF6739	1:200 (western blot)
Antibody	Anti-Vangl2 (rat monoclonal)	Millipore	Cat #MABN750	1:100
Antibody	Anti-E-cadherin, clone DECMA-1 (rat monoclonal)	ThermoFisher	Cat #14–3249-82	1:1000
Antibody	Anti-Frizzled6 (Goat polyclonal)	R&D Biosystems	Cat #AF1526	1:400
Antibody	Anti-GFP (chicken)	Abcam	Cat # ab13970	1:2000 (keratinocytes)
Antibody	Anti-Myc (rabbit)	Sigma	Cat #C3956	1:2000 (keratinocytes)
Antibody	Anti-FLAG (mouse)	Stratagene	Cat # 200472–21	1:2000 (keratinocytes)
Antibody	Anti-Guinea Pig, Alexa Fluor 488 (Goat)	Invitrogen	Cat #A11073	1:2000
Antibody	Anti-Guinea Pig, Alexa Fluor 647 (Donkey)	Invitrogen	Cat #A21450	1:2000
Antibody	Anti-Chicken, Alexa Fluor 488 (Goat)	Invitrogen	Cat #A-11039	1:2000
Antibody	Anti-Chicken, Alexa Fluor 488 (Donkey)	Jackson ImmunoResearch	Cat #703–545-155	1:2000
Antibody	Anti-Rabbit, Alexa Fluor 555 (Donkey)	Invitrogen	Cat #A-31572	1:2000
Antibody	Anti-Rabbit, Alexa Fluor 488 (Donkey)	Jackson ImmunoResearch	Cat #711–545-152	1:2000
Antibody	Anti-Rat, Alexa Fluor 647 (Donkey)	Jackson ImmunoResearch	Cat #712–605-153	1:2000
Antibody	Anti-Rat, Alexa Fluor 555 (Goat)	Invitrogen	Cat #A-21434	1:2000
Antibody	Anti-Rat, Alexa Fluor 488 (Donkey)	Invitrogen	Cat #A-21208	1:2000
Antibody	Anti-Goat, Alexa Fluor 647 (Donkey)	Jackson ImmunoResearch	Cat #705–605-147	1:2000
Antibody	Anti-rabbit IRDye 680CW (Goat)	LI-COR	Cat # 926–68073	1:10000
Antibody	Anti-rat IRDye 800CW (Goat)	LI-COR	Cat # 925–32219	1:10000
Antibody	Anti-goat IRDye 800CW (Goat)	LI-COR	Cat # 926–68074	1:10000
Plasmid	Celsr1-GFP	Devenport and Fuchs (2008)		full length WT Celsr1 in pEGFPN1
Plasmid	Celsr2-GFP	Tadeshi Uemura		full length WT Celsr2 in pEGFPN3
Plasmid	pCMV-Celsr13xflag	Devenport and Fuchs (2008)		full length WT Celsr1 in Stratgene pCMV-3Tag-8
Plasmid	pCMV-Celsr1-3xmyc	Devenport and Fuchs (2008)		full length WT Celsr1 in Stratgene pCMV-3Tag-9
Plasmid	K14-Fz6-tdTomato	Heck and Devenport (2017)		Full length Fz6 tagged to tdTomato in the C-terminus
Plasmid	tdTomato-Vangl2	Stahley et al. (2021)	Heck and Devenport, (2017)	Full length Vangl2 tagged to tdTomato in the N-terminus
Plasmid	pT2Aneo-tdTomato-CAAX	Addgene	Cat# 170284	tdTomato with CAAX motif
Plasmid	EGFP-CAAX	Addgene	Cat# 86056	GFP with CAAX motif
Software, algorithm	Matlab	MathWorks		
Software, algorithm	Tissue Analyzer; Packing analyzer	Aigouy et al. (2010), Aigouy et al. (2016)		
Software, algorithm	ImageJ/Fiji			
Software, algorithm	Graphpad Prism			

### Immunofluorescence and image acquisition of embryonic backskins

Fixing and staining of backskins was done as previously described (Basta et al., 2021). Briefly, E15.5 embryos were fixed in 4% paraformaldehyde in PBS ++ for 1 h at room temperature. For all antibodies apart from P-cadherin, backskins were dissected and blocked at 4°C in 2% normal goat serum, 2% normal donkey serum (or 4% normal donkey serum and no goat serum when staining for Fz6), 1% bovine serum albumin and 1% fish gelatin in PBT2 (PBS with 0.2% Triton X-100). For P-cadherin, samples were blocked in in 2% normal goat serum, 2% normal donkey serum, 1% bovine serum albumin and 1% fish gelatin in TBT2 (TBS with 0.2% Triton X-100). Following incubation in block, samples were incubated in primary antibody in PBT2 block (TBT2 block for P-cadherin staining) at 4°C overnight. Samples were washed in PBT2 five times for at least 30 min at room temperature, incubated with secondary antibodies and Hoechst (Invitrogen, Cat: H1399, 1:1000) overnight at 4°C, and then washed in PBT2 three times for at least 10 min. After a final wash in PBS, samples were mounted in Prolong Gold.

The following primary antibodies were used: guinea pig anti-Celsr1 (Danelle Devenport, 1:1000), rabbit anti-E-cadherin (1:250, Cell Signaling: 3195), rat anti-Vangl2 (1:100, Millipore, Cat: MABN750), goat anti-Fz6 (1:400, R&D Biosystems, Cat: AF1526), rabbit anti-Sox9 (Millipore, AB5535, 1:1000), rat anti-P-cadherin (Clontech, M109, 1:200). Alexa Fluor −488, −555, and −647 secondary antibodies were used at 1:2000 (Invitrogen or Jackson ImmunoResearch). See Table 2 for full list of antibodies and reagents.

For hair follicle polarity analysis, images were acquired using a Nikon A1R-Si confocal microscope operated by NIS Elements software, using PlanApo 20 × 0.75 NA air and 60 × 1.4 NA oil immersion objectives for resonance and galvo image capture respectively. 20 × images were then stitched in NIS Elements and processed in Fiji to generate an Average Intensity Projection (AIP) for input to the automated segmentation and angle calculation MATLAB algorithm. 60 × images were processed in Fiji.

For analysis of core PCP protein asymmetry in the basal layer, images were acquired on Nikon A1R-Si confocal microscope controlled by NIS Elements software using PlanApo 60 × 1.4 NA oil. Images were processed using NIS elements and ImageJ.

### Isolation of primary keratinocytes and keratinocyte culture

Keratinocytes were isolated from *Celsr1* ^*−/−*^ *; Celsr2* ^*−/−*^ pups at P1 and established as cell lines using previously published protocol (Nowak and Fuchs, 2009). Keratinocytes were grown using E-Media prepared in the laboratory according to published protocol (Nowak and Fuchs, 2009) supplemented with 50 µM calcium chloride. For live FRAP experiments, phenol-red free DMEM and F-12 were used to prepare pigment-free imaging E-media. Cells were transfected using Effectene reagent following a modified manufacturer’s protocol. 300 ng of DNA was used in the transfection mix for each well of 12-well plates and 400 ng plasmid DNA was used for each well of 6-well plates and 35-mm imaging dishes. For co-transfection with two different plasmids, one of which is Celsr1-GFP or Celsr2-GFP, a ratio of 2:1 of Celsr1/Celsr2: the other plasmid DNA was used. See Table 2 for full list of plasmids used in this study.

For junction enrichment assays, approximately 100,000 keratinocytes were seeded onto fibronectin coated 1.8-mm, #1.5 glass coverslips in each well of 12-well plates. Approximately 24 h post-seeding, cells were transfected with the necessary plasmids. 24 h post transfection, cells were switched from low calcium E-media (50 µM) to high calcium E-media containing 1.5 mM Ca^2+^. After 24 h of incubation, cells were fixed and stained for imaging (see below). For heterotypic junction enrichment assays, −150,000 cells were seeded into each well of 6 well plates. 24 h post seeding, each well was individually transfected with one of the following constructs: Celsr1-3XFLAG, Celsr1-3X-Myc, Celsr1-GFP or Celsr2-GFP. 24 h post-transfection, cells were trypsinized, mixed and replated, with one Celsr1-FLAG or Celsr1-Myc transfected well combined with either one well of Celsr1-GFP or one well of Celsr2-GFP. −180,000 cells from the 1:1 mixture were seeded onto fibronectin coated 1.8-mm, #1.5 glass coverslips in each well of 12-well plates. 4–5 h post plating, media was switched to E-media containing 1.5 mM Ca^2+^. After an additional 24 h, cells were fixed and stained for microscopy.

### Immunofluorescence of keratinocytes

After incubation in high calcium E-media, confluent monolayers of keratinocytes were rinsed in PBS containing calcium and magnesium (PBS++) and fixed with 4% PFA prepared in PBS++ for 10 min at room temperature, followed by permeabilization for 10 min in PBS containing 0.1% Triton-X 100 (PBT1). Primary antibodies were diluted 1:2000 in PBT1 and cells were incubated with the same for 30 min. Following primary antibody treatment, cells were washed three times in PBS and further incubated for 30 min in secondary antibodies and Hoechst diluted to 1:2000 in PBS. Cells were finally washed three times in PBS and mounted on glass slides using Prolong Gold, cured overnight in the dark and imaged.

### Image segmentation and polarity analysis

*S*egmentation of basal epidermal cells and polarity analysis of core PCP proteins. Cell Pose (Stringer et al., 2021) was used to segment images of the whole mount embryonic epidermis. Segmentation masks were obtained using the E-cadherin or P-cadherin markers, and masks were post-processed, and hand corrected in ImageJ. The same mask from the E-cadherin or P-cadherin marker was used on the other channels in the image.

As previously described, polarity analysis was done using the Tissue Analyzer V2 software in ImageJ (Aigouy et al., 2010; Basta et al., 2021). Cell edges were defined by the segmentation masks generated as described above. Tissue Analyzer used the segmentation masks to calculate the axis and magnitude (nematic order) of membrane localized proteins (as defined in Aigouy et al., 2016), including for PCP proteins. Circular histograms plotting the data were generated using MATLAB, with average polarity magnitude indicating the angle and strength of polarity. Prior to analysis, images were rotated to align them with the anterior-posterior axis.

Segmentation and polarity analysis of hair follicles. To determine the orientation of hair follicle growth, AIP images were analyzed using a custom MATLAB script followed by visual *ad hoc* hand correction. This script segments regions of the hair follicle based on Sox9 and P-cadherin fluorescence intensity and uses the geometric relationship between segmented regions to categorize polarized and non-polarized follicles as well as to calculate the angle of growth of polarized follicles.

### Image acquisition and analysis of junction enrichment assay

Cells were imaged using a PlanApo 20 × 0.75NA Air objective with additional zoom on a Nikon A1R-Si confocal microscope using the relevant combination of 405, 488, 561, and 643 nm laser illumination. Image acquisition was sequentially carried out to avoid bleed-through. Maximum intensity projections from Z stacks were made in Fiji. Images were background subtracted. ROIs were drawn along the junctions marked by either Celsr1-GFP/Celsr2-GFP/GFP-CAAX as applicable. Another ROI was made along the periphery of the participating cell or the two adjacent cells sharing the junction, as applicable. A ratio was obtained of the background corrected mean intensities of the junction and the cell pair/individual cell ROI as follows:

$$\text{Junction}\;\text{enrichment}=\frac{\text{border}\;\text{mean}\;\text{intensity}}{\text{cell}\;\text{or}\;\text{cell}\;\text{pair}\;\text{mean}\;\text{intensity}}$$

### FRAP

Approximately 150,000 keratinocytes were seeded in #1.5 glass bottom dishes (ibidi #81151) coated with fibronectin. 20–24 h post-plating, cells were transiently transfected with Celsr1-GFP or Celsr2-GFP plasmids. 24 h post-transfection, cells were switched to E-media containing 1.5 mM Ca^2+^ and incubated for an additional 20–24 h for Celsr1-GFP and Celsr2-GFP expression. Before imaging, cells were switched to phenol-red free E-media containing 1.5 mM Ca^2+^ and 20 mM HEPES. Cells were imaged using a 488 nm laser, Plan Apo 60 × 1.4NA oil immersion objective (with additional zoom that rendered a pixel size of 110 nm) on Nikon A1R confocal microscope equipped with a stage-top Tokai-Hit incubation chamber to maintain 37° and 5% CO_2_. Keeping magnification, laser power (both for bleach and acquisition), pixel dwell time and acquisition rate constant across all measurements, 1-um diameter circular bleach ROIs and three ROIs per image were created to sample the junction(s) or cells edge(s). The FRAP acquisition sequence consisted of three reference pre-bleach images followed by bleach (5.9 s) and finally 60 frames with 5-s intervals to monitor fluorescence recovery. The acquired images in the time series were checked for any Z-drift and corrected for XY-drift in Fiji. A reference ROI was made in a non-bleached region to correct for overall bleaching during image acquisition. A background ROI was created outside the cell in each image. The ROI values were extracted from drift corrected images in NIS elements software for subsequent analysis in Microsoft Excel and Graphpad Prism. Each image time series was background and bleach corrected (to be referred as corrected intensity henceforth) and thereafter the corrected intensity profile was normalized as *(F*_*t*_ *–F*_*bleach*_ *)/(F*_*ini*_ *–F*_*bleach*_ *)*, where, F_t_ is the corrected intensity of the ROI at a given time point, F_bleach_ is the corrected intensity at the time point immediately after bleaching, F_ini_ is the mean ROI intensity of the three pre-bleach frames. Each mean recovery curve was fitted to exponential one phase association equation in Graphpad Prism and the fitted Plateau and Y_0_ values were used to determine the *immobile fraction* = *1- { (Plateau-Y*_*0*_ *)/(1- Y*_*0*_ *) }*. The averaged traces for each condition was fitted to the model with an r-squared value >0.93.

Data was analyzed using Nikon NIS elements and ImageJ/FIJI and Microsoft Excel. Graphs were plotted using Graphpad Prism. Data represented is pooled from at least two independent experiments for cell edges and at least three independent experiments for junctions where each experiment follows the represented trend.

### Statistics

Details related to sample size, error bars and statistical significance are described in the legends for each figure. Differences between distributions of junction enrichment ratios and cell mean intensities were tested by non-parametric KS test using Graphpad Prism software.

## Data Availability

The raw data supporting the conclusion of this article will be made available by the authors, without undue reservation.
