# Supplementary material for: Celsr1 and Celsr2 exhibit distinct adhesive interactions and contributions to planar cell polarity
Source: Front Cell Dev Biol. 2023 Jan 12;10:1064907. doi: 10.3389/fcell.2022.1064907 (PMC9878842; doi:10.3389/fcell.2022.1064907)
Supplement: Supplementary file 1 [file Image1.pdf]

Figure 1 - Supplement

A Celsr1 Genomic Region

Chr15:85783130-85918404 bp, Reverse strand ←

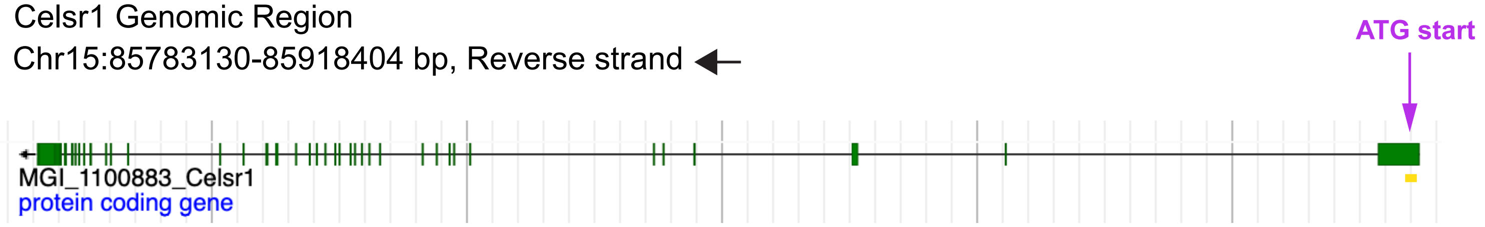

B Celsr1 targeted alelle

81 bp Deletion (highlighted), **ATG start site + signal sequence**

5’ CCCCGGCCGCGCCGGGTGGATGCGGAGGTGCGCCGGGTGCATGGATTGTGCTCCGGCGCCCGGCTCGGGCTGCG
GCTCCGGGCC**ATGGCGCCGTCATCGCCGCGCGTGCTGCCCGCGCTGGTGTTGCTGGCCGCCGCCGCCCTGCCAGC**
CCTGGAGTTGGGAGCAGCCGCTTGGGAGCTGCGGGTGCCTGGCGGGGCCCGCGCCTTCGCTCTCGGACCCGGCT 3’

C

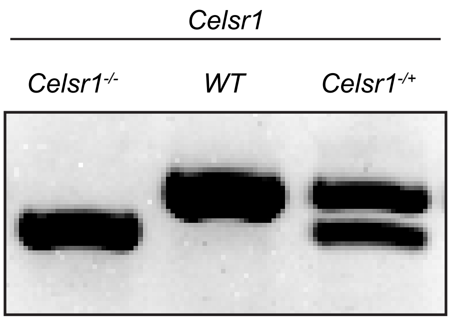

D Celsr2 Genomic Region

Chr3:108298167-108323383 bp, Reverse strand ←

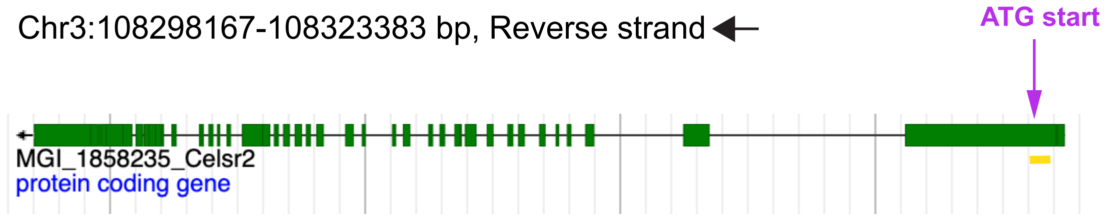

E Celsr2 targeted alelle

81 bp Deletion (highlighted), **ATG start site + signal sequence**

GGCGGGGCCGGCAGGAGCCGGAGGAAGAGCCGCCGCGGCTGTTGACCCGGCTGGCCGGGAACAGGGAGAG**ATG**
CGGAGCCGGGCTGCCAGCGCCCCCTCCCAACGCCGCTGCTGCCGCTGCTACTGCTGTTGCTGCTGCTGCCACCG
TCGCCACTACTGGGAGATCAAGTGGGGCCCTGTCGTTCTCTGGGATCCGGGGGACGCAGCTCCTCTGGGGCCTGCG

F

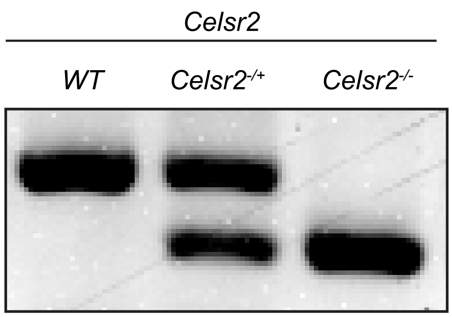

Supplement to Figure 1. (A) Celsr1 genomic region on chromosome 15, reverse strand. ATG start site position (magenta arrow) and 81 bp deletion flanking the ATG (yellow bar) are shown (image credit, MGI – JBrowse). **(B)** CRISPR/Cas9 targeted *Celsr1* allele. The ATG and signal sequence (magenta font) and 81 bp deleted sequence (yellow highlight) are shown. Genotyping primers surround the deletion site. **(C)** Representative images of genotyping PCR results for $Celsr1^{<em1Dev>}$. WT PCR product is 396bp (second lane), while knockout PCR product is 315 bp (first lane). Heterozygous animals give PCR products of both sizes (third lane). **(D)** Celsr2 genomic region on chromosome 3, reverse strand. ATG start site position (magenta arrow) and 81 bp deletion flanking the ATG (yellow bar) are shown (image credit, MGI – JBrowse). **(E)** CRISPR/Cas9 targeted *Celsr2* allele. The ATG and signal sequence (magenta font) and 81 bp deleted sequence (yellow highlight) are shown. Genotyping primers surround the deletion site. **(E)** Representative images of genotyping PCR results for $Celsr2^{<em1Dev>}$. WT PCR product is 350 bp (first lane), while knockout PCR product is 269 bp (third lane). Heterozygous animals give PCR products of both sizes (second lane).

Figure 4 - Supplement

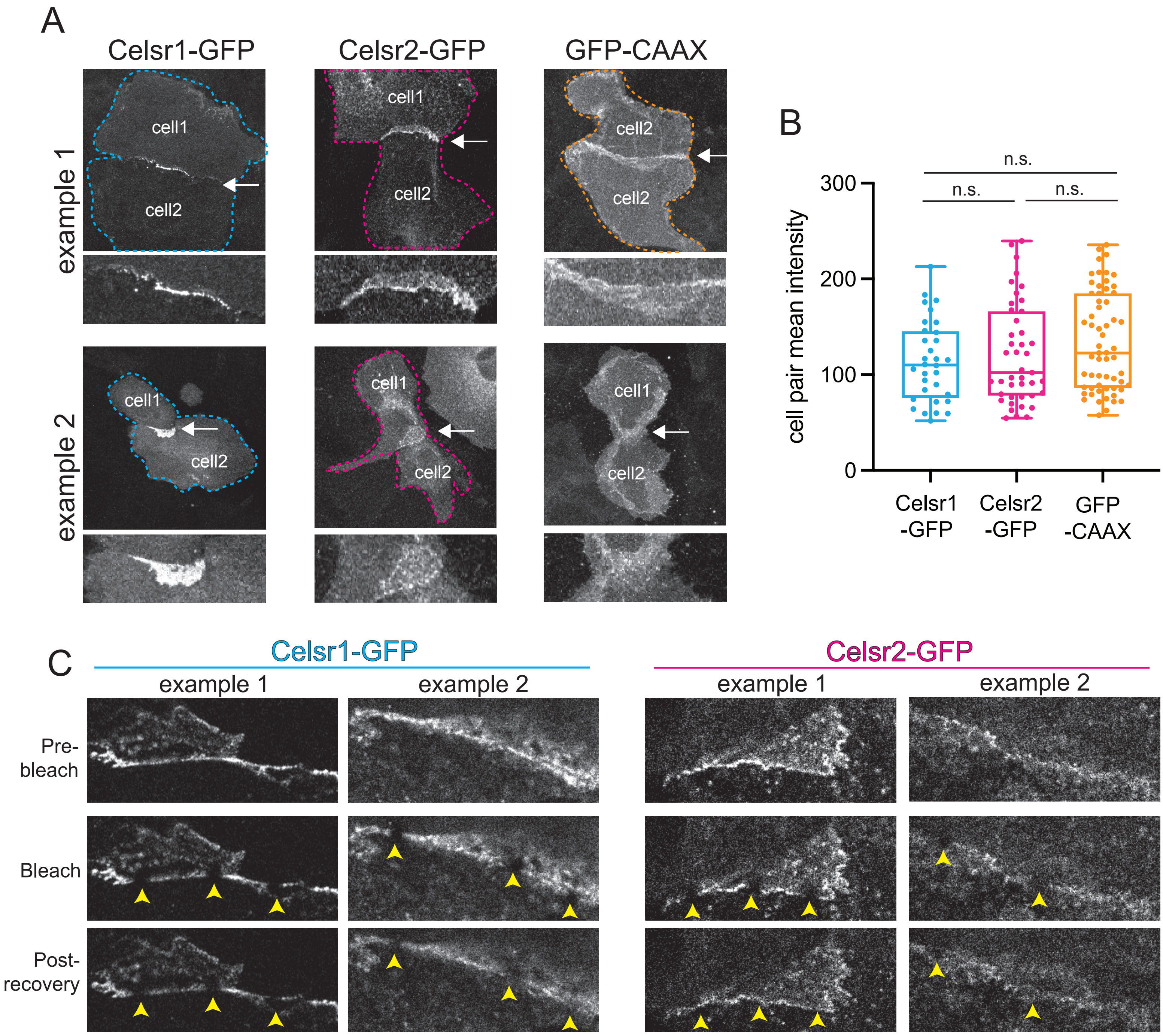

Supplement to Figure 4. Junctional enrichment and stability of Celsr1 and Celsr2. (A) Additional representative images of cell pairs expressing Celsr1-GFP, Celsr2-GFP or GFP-CAAX as indicated. Bottom panels show zoomed in junctional regions. **(B)** Plot of the total mean intensity of cell pairs expressing Celsr1-GFP, Celsr2-GFP or GFP-CAAX. Note that the overall fluorescence intensity of cell pairs transfected with each construct is not significantly different, indicating that differences in expression levels does not account for the differences in junctional enrichment observed (see Figure 4B). n=32 Celsr1-GFP junctions, n=43 Celsr2-GFP junctions, n=60 GFP-CAAX junctions. Kolmogorov-Smirnov tests $p > 0.1$. **(C)** Fluorescence Recovery After Photobleaching (FRAP) of junctional Celsr1-GFP and Celsr2-GFP. Shown are additional representative images of the junctional region between cell pairs expressing Celsr1-GFP or Celsr2-GFP before and after bleaching, as indicated. Bleached ROIs are marked by yellow arrowheads.

Figure 5 - Supplement

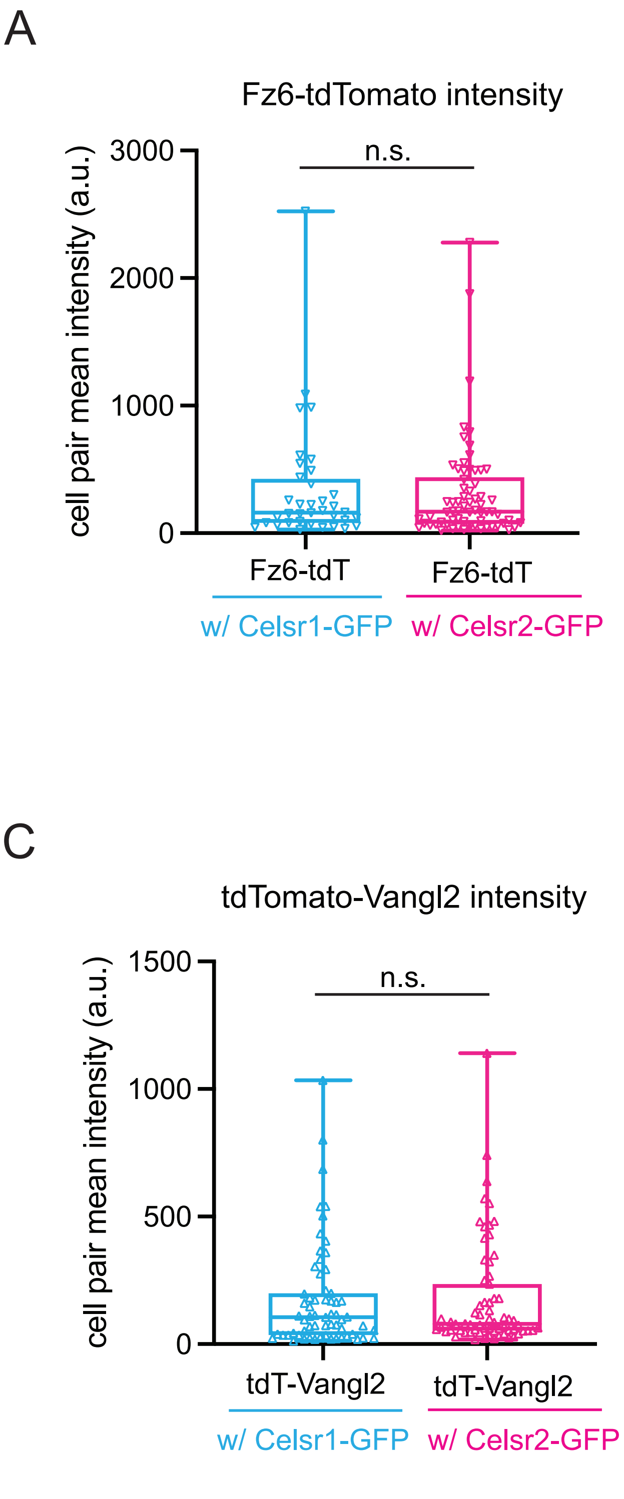

Supplement to Figure 5. Differential recruitment of Fz6 and Vangl2 to junctions by Celsr1 and Celsr2 is not due to differences in expression levels of transfected cells. (A) Mean fluorescence intensity of total Fz6-tdTomato in cell pairs co-transfected with Celsr1-GFP or Celsr2-GFP. **(B)** Mean fluorescence intensity of total Celsr1-GFP or Celsr2-GFP in cell pairs co-transfected with Fz6-tdTomato. **(C)** Mean fluorescence intensity of total tdTomato-Vangl2 in cell pairs co-transfected with Celsr1-GFP or Celsr2-GFP. **(D)** Mean fluorescence intensity of total Celsr1-GFP or Celsr2-GFP in cell pairs co-transfected with tdTomato-Vangl2. Data pooled from two independent experiments for Fz6-tdTomato and three independent experiments for tdTomato-Vangl2. Kolmogorov-Smirnov tests, $p > 0.1$.
